# Probiotic Lactobacilli Limit Avian Influenza Virus Subtype H9N2 Replication in Chicken Cecal Tonsil Mononuclear Cells

**DOI:** 10.3390/vaccines8040605

**Published:** 2020-10-13

**Authors:** Nadiyah Alqazlan, Mohammadali Alizadeh, Nitish Boodhoo, Khaled Taha-Abdelaziz, Eva Nagy, Byram Bridle, Shayan Sharif

**Affiliations:** 1 Department of Pathobiology, Ontario Veterinary College, University of Guelph, Guelph, ON N1G 2W1, Canada; nalqazla@uoguelph.ca (N.A.); alizadem@uoguelph.ca (M.A.); boodhoon@uoguelph.ca (N.B.); abdelazk@uoguelph.ca (K.T.-A.); enagy@ovc.uoguelph.ca (E.N.); bbridle@uoguelph.ca (B.B.); 2 Pathology Department, Faculty of Veterinary Medicine, Beni-Suef University, Beni-Suef 62511, Egypt

**Keywords:** probiotics, lactobacilli, immunity, chicken, cecal tonsil, CpG ODN, avian influenza virus, H9N2

## Abstract

Low pathogenic avian influenza virus (LPAIV) H9N2 poses significant threat to animal and human health. The growing interest in beneficial effects of probiotic bacteria on host immune system has led to research efforts studying their interaction with cells of host immune system. However, the role of lactobacilli in inducing antiviral responses in lymphoid tissue cells requires further investigation. The objective of the present study was to examine the antiviral and immunostimulatory effects of lactobacilli bacteria on chicken cecal tonsils (CT) cells against H9N2 LPAIV. CT mononuclear cells were stimulated with probiotic *Lactobacillus spp* mixture either alone or in combination with a Toll-like receptor (TLR)21 ligand, CpG oligodeoxynucleotides (CpG). Pre-treatment of CT cells with probiotic lactobacilli, alone or in combination with CpG, significantly reduced H9N2 LPAIV replication. Furthermore, lactobacilli alone elicited cytokine expression, including IL-2, IFN-γ, IL-1β, IL-6, and IL-12, and IL-10, while when combined with CpG, a significantly higher expression of (interferon-stimulated gene (viperin)), IL-12, IL-6, CXCLi2, and IL-1β was observed. However, none of these treatments induced significant changes in nitric oxide production by CT cells. In conclusion, probiotic lactobacilli demonstrated a modulatory effect on CT cells, and this correlated with enhanced antiviral immunity and reduced H9N2 LPAIV viral replication.

## 1. Introduction

Influenza A virus (IAV) is an enveloped virus with a segmented, single-stranded, negative-sense RNA genome in the family *Orthomyxoviridae*. Infection of chickens with avian influenza virus (AIV) is characterized by the presence and replication of infectious virus particles in the upper respiratory and gastrointestinal tracts [1,2]. Avian influenza viruses of the H9 subtype, which are classified as low-pathogenic avian influenza viruses (LPAIV), cause mild disease and low mortality in poultry compared to highly pathogenic avian influenza viruses (HPAIV). The emergence of H9 subtype viruses in poultry constitutes a public health risk because of their potential of crossing the species barrier and infecting humans, as evident in recent reports of H9N2 infections in humans across Asia and Africa [3,4,5]. These reported infections in humans show potential for H9 viruses to adapt to humans and could, therefore, trigger a pandemic. Aside from human and animal health concerns, H9N2 viruses are associated with reduced growth rates in broilers and egg production in breeders and layers [6,7], signifying the importance of these viruses. Thus, on-farm control of AIV via vaccination or feed additives will not only mitigate the economic burden on poultry production, but it will also reduce the public health risk caused by this virus.

There are vaccines available against H9 AIV; however, these vaccines are not very effective, partly because of antigenic variants of the virus [8]. Low pathogenic avian influenza viruses are able to replicate in the gastrointestinal tract. Therefore, it is essential to understand how these viruses interact with their host’s gut-associated immune system [9]. Several studies have demonstrated the presence of infectious H9 AIV subtypes in cecal tonsil (CT) cell-derived homogenates [10,11,12]. Additionally, the hemagglutinin (HA) and matrix (M) genes of H9N2 LPAIV have been detected in CT using quantitative real-time PCR (qRT-PCR), while the presence of viral nuclear protein (NP) of H7 LPAIV in CT was confirmed using immunohistochemistry [13,14,15]. However, systemic viral dissemination is not restricted to H9N2, as H5N1 HPAIV has also been detected in CT cell lysate. Moreover, an H5N6 AIV, originating from wild birds, has been demonstrated to replicate in chicken CT cells, and an H7N3 HPAIV was associated with high histology and immunohistochemistry scores in chicken CT samples [16,17]. These AIV subtypes have the potential to cause a systemic infection similar to HPAIV. Therefore, it is conceivable that induction of innate responses in CT could limit viral replication, thereby reducing viral shedding and transmission.

Interaction of gut-associated lymphoid tissues (GALT) with various foodborne antigens, gut microbiota, and pathogens plays a major role in the development and modulation of local and systemic immune responses [18,19,20]. Conserved pattern recognition receptors (PRR) mediate the innate responses mounted by CT [21]. The best characterized PRRs are the Toll-like receptors (TLRs), which are transmembrane-bound as well as intracellular receptors expressed by various leukocytes of mammals and birds. TLRs can selectively recognize pathogen-associated molecular patterns [22]. CpG oligodeoxynucleotides (ODN) 2007, a TLR21 ligand, has shown potential as a prophylactic and therapeutic agent against AIV infection in vitro, in ovo, and in vivo [21,23,24,25,26,27], as well as an adjuvant [28,29]. 

Beyond the use of TLR ligands, treatment with lactic acid-producing bacteria (LAB) has been shown to modulate the host response in ovo, in vivo, and in vitro [18,30,31,32,33,34,35,36]. In fact, substantial research efforts have focused on unravelling the beneficial role of LAB, particularly *Lactobacillus* species in growth performance and infection control. The immunomodulatory activities of probiotic bacteria lie, in part, in their ability to induce cytokine production, which in turn leads to regulation of innate and adaptive immune responses. Recent evidence indicates that species and strains of bacteria used as probiotics have differential effects on host responses [18,37]. Probiotic LAB used in chickens have been shown to induce intestinal mucosal immunity [38], in addition to a systemic immune response characterized by an increase in total serum antibody titers and a significant alteration in expression of various immune system genes associated with splenic B and T cells [18,31,32,33,39,40], which can lead to protection of chickens against AIV [41]. Despite our current understanding of CT cell responses, immune responses induced in CT cells to AIV infection require a deeper understanding.

Previous studies have demonstrated that CT cells are capable of mounting a robust immune response, following exposure to probiotic lactobacilli and TLR ligands such as CpG ODN [18,21]. However, there is a lack of knowledge on the role of these mounted responses in conferring protection against viral infections, specifically AIV. Therefore, the objective of the current study is to determine the infectivity of the H9N2 AIV in chicken cecal tonsil cells and to examine the potential of probiotic lactobacilli to induce effective antiviral and inflammatory responses and limit viral replication in these cells in vitro. 

## 2. Materials and Methods

### 2.1. Experimental Animals

Twenty-four one-day-old specific pathogen-free (SPF) White Leghorn layers were purchased from the Canadian Food Inspection Agency (Ottawa, ON, Canada). Birds were grouped, housed, and provided with ad libitum food and water in the isolation facility of the Ontario Veterinary College at the University of Guelph, Canada. All experimental procedures were approved by the University of Guelph Animal Care Committee and conducted according to specifications of the Canadian Council on Animal Care under Animal Utilization Protocol number 4203, and all experiments were carried out in 2019–2020.

### 2.2. Preparation of Cecal Tonsil Mononuclear Cells

Whole CTs were aseptically collected from three-week-old layers and stored on ice in phosphate-buffered saline (PBS) supplemented with penicillin (10 U/mL) and streptomycin (10 µg/mL). CT tissue samples were cut open, washed three times with supplemented PBS and then cut into fine slices. Tissue samples were subsequently digested with collagenase 1 (Sigma-Aldrich, Oakville, ON, Canada); 80 U/mL at 37 °C for 20 min) in supplemented PBS. Whole tissue digest was applied onto 40-μm cell strainers (BD Biosciences, San Jose, CA, USA), and crushed using the flat end of a 10-mL syringe plunger. The CT cell suspension was prepared by layering (2:1) onto histopaque 1077 (Sigma-Aldrich, Oakville, ON, Canada) density-gradient and centrifuged at 400× *g* for 20 min to allow the separation of mononuclear cells. Mononuclear cells were subsequently aspirated from the interface and washed at 400× *g* for 5 min in RPMI 1640 (Gibco^®^, Life Technologies, Burlington, NJ, USA) with penicillin (10 U/mL), and streptomycin (10 μg/mL). Mononuclear cells were suspended in complete RPMI cell culture medium; RPMI 1640 medium containing 0.2% bovine serum albumin, 25 mM HEPES (Gibco^®^, Life Technologies, Burlington, NJ, USA), penicillin (10 U/mL), and streptomycin (10 μg/mL). Cell number and viability were assessed using a hemocytometer and trypan blue exclusion method. Mononuclear cells were suspended in complete RPMI cell culture medium at a density of 1 × 10^7^ cells/mL. CT mononuclear cells from eight chickens were prepared for each subsequent experiment, including infection with AIV, evaluation of antiviral activity, and quantification of gene expression and nitric oxide production.

### 2.3. Probiotic Lactobacillus Species, Media, and Growth Conditions

In this study, a cocktail of three avian *Lactobacillus* species was used, including *L. salivairus, L. johnsonii, and L. reuteri* [18,31,34]. Bacteria were grown separately and anaerobically in De Man, Rogosa and Sharpe (MRS) broth (Becton Dickinson, Mississauga, ON, Canada) at 37 °C without shaking for 24 h and sub-cultured twice. Bacteria were harvested by centrifugation (3000× *g* for 10 min) at the beginning of the stationary growth phase (six hours post-inoculation). The pelleted bacteria were then washed three times in PBS and suspended in PBS. Heat-inactivation of lactobacilli was performed in a water bath at 100 °C for 30 min. Inactivation was confirmed by the absence of colony formation on MRS agar plates.

### 2.4. TLR Ligands

A synthetic class B CpG ODN 2007 was used as a TLR21 ligand [5′-TCGTCGTTGTCGTTTTGTCGTT-3′] (InvivoGen, Vista Sorrento Pkwy San Diego, CA, USA) with a phosphorothioate backbone (CpG). The CpG was reconstituted in endotoxin-free water and diluted to a working concentration of 5 µg/mL using RPMI 1640 medium-based. This concentration was previously shown to be an optimal concentration [21].

### 2.5. Virus Strain

A LPAIV, A/TK/IT/13VIR1864-45/2013 (H9N2), was used in this study. The virus was propagated in 10-day-old embryonated chicken eggs for 72 h at 37 °C. Embryos were monitored every 24 h, and dead embryos were discarded. After incubation, eggs were refrigerated at 4 °C overnight. Then allantoic fluid was collected and centrifuged at 400× *g* for five min and then stored at −80 °C until further use. Viral titers were determined based on 50% tissue culture infectious dose (TCID_50_) by using Madin-Darby canine kidney (MDCK) cells. 

### 2.6. Infection of CT Cells with H9N2 AIV

CT mononuclear cells (*n* = 8 biological replicates) (10^6^ cells/well in 100 µL of RPMI medium) were infected with H9N2 AIV at a multiplicity of infection (MOI) of one for one hour at 41 °C and 5% CO_2_. In a pilot experiment, various MOI were tested (0.01, 0.1, and 1) to determine the optimal MOI for H9N2 AIV replication in CT mononuclear cells. Plates were then centrifuged, and the supernatant was discarded. The mononuclear cells were washed with media and centrifuged again to remove virus residue. Subsequently, mononuclear cells were suspended in fresh complete RPMI 1640 medium containing 2 µg/mL of L-1-tosylamido-2-phenylethyl chloromethyl ketone (TPCK)-treated trypsin (Sigma-Aldrich; Cat. No. T1426). One well was left uninfected for each time point and each biological replicate. Mononuclear cells were incubated at 41 °C in a humidified 5% CO_2_ environment for 0, 6, 12, 18, 24, and 30 h post-infection (hpi). Cell culture supernatants at each time point were collected and stored at −80 °C for virus titration.

### 2.7. Stimulation of CT Cells with a Cocktail of Probiotic Lactobacilli Cocktail and/or CpG ODN 2007

For each of the samples (*n* = 8 biological replicates), 100 µL of the mononuclear cell suspension (10^6^ cells/well) were seeded into a 96-well round-bottom plate. CT mononuclear cells were stimulated with 5 µg/mL of CpG, 10^7^ colony-forming units (CFU) of probiotic lactobacilli cocktail (PROB), or 10^8^ CFU of PROB alone or a combination of 5 µg/mL of CpG and 10^7^ or 10^8^ CFU of PROB. Treatment with PBS was used as a negative control for each time point. The CT mononuclear cells were subsequently incubated at 41 °C in a humidified 5% CO_2_ environment. At 3, 6, and 24 h post-stimulation (hps) mononuclear cells were harvested and lysed in TRIzol^®^ for RNA isolation (Invitrogen, Carlsbad, CA, USA). Because of the small number of cells isolated from cecal tonsils of individual birds and low RNA yield, RNA samples were pooled, resulting in a total of four biological replicates per treatment group at each time point, which were subsequently used for qRT-PCR analysis (Table 1). Supernatants of stimulated CT mononuclear cells were collected at 3, 6, and 24 hps for quantification of NO using the Griess assay, according to the manufacturer’s protocol (Promega, Madison, WI, USA).

### 2.8. Evaluation of Antiviral Activity of CT Mononuclear Cells Stimulated with Lactobacilli and/or CpG ODN 2007

CT mononuclear cells from eight chickens (10^6^ cells/ well in 100 µL of RPMI medium) were stimulated as described above. Following 24 h of stimulation, supernatants were discarded, and mononuclear cells were infected at a MOI of one with H9N2 AIV for one hour at 41 °C and 5% CO_2_. Mononuclear cells were then washed and resuspended in complete RPMI 1640 medium containing 2 µg/mL of TPCK-treated trypsin. Mononuclear cells were subsequently incubated at 41 °C in a humidified 5% CO_2_ environment, and the supernatants were collected, 6 hpi, and stored at −80 °C for later virus titration.

### 2.9. RNA Extraction and Complementary Single-Stranded DNA (cDNA) Synthesis

Total RNA was extracted from the CT mononuclear cells using TRIzol^®^ according to the manufacturer’s protocol with the addition of 10 µg of glycogen (Thermo Scientific, Life Technologies, Burlington, NJ, USA) as a carrier to increase recovery of RNA. Total RNA was treated with a DNA-free™ Kit for DNA removal (Ambion, Carlsbad, CA, USA) and quantified using a NanoDrop spectrophotometer (Thermo Scientific, Wilmington, DE, USA). Subsequently, 500 ng of purified RNA was reverse transcribed to cDNA using a Superscript^®^ II First Strand Synthesis kit (Invitrogen) and oligo-dT primers, according to the manufacturer’s recommended protocol. The resulting cDNA was diluted 1:10 in diethyl pyrocarbonate (DEPC)-treated water.

Total RNA was extracted from the CT mononuclear cells using TRIzol^®^ according to the manufacturer’s protocol with the addition of 10 µg of glycogen (Thermo Scientific, Life Technologies, Burlington, ON, Canada) as a carrier to increase recovery of RNA. Total RNA was treated with a DNA-free™ Kit for DNA removal (Ambion, Carlsbad, CA, USA) and quantified using a NanoDrop spectrophotometer (Thermo Scientific, Wilmington, DE, USA). Subsequently, 500 ng of purified RNA was reverse transcribed to cDNA using a Superscript^®^ II First Strand Synthesis kit (Invitrogen) and oligo-dT primers, according to the manufacturer’s recommended protocol. The resulting cDNA was diluted 1:10 in diethyl pyrocarbonate (DEPC)-treated water.

### 2.10. Quantitative Real-Time PCR

A qRT-PCR using SYBR Green (Roche) was performed on diluted (1:10) cDNA using the LightCycler 480 II (Roche Diagnostics GmbH, Mannheim, Germany) as previously described with some modifications [18]. Briefly, the amplification conditions consisted of pre-incubation for 10 min at 94 °C, followed by 45 cycles of 95 °C for 10 s, 55–64 °C annealing as described in Table 1 for each of the primers for 5 s and elongation and signal acquisition (single mode) at 72 °C for 10 s. Subsequent melting curve analysis was performed by heating to 95 °C for 10 s, cooling to 65 °C for 1 min, and heating to 97 °C. Primers were synthesized by Sigma Aldrich. Specific sequences of primers are listed in Table 1.

### 2.11. Statistical Analyses

The expression of genes was calculated relative to the housekeeping gene β-actin using software on the LightCycler^®^ 480 II (Roche Diagnostics GmbH). Data represent the mean of four biological replicates ± standard error of the mean. Data analysis of multiple treatments for virus replication in CT cells (between time points) and gene expression (between different treatments within a time point) was performed with one-way analysis of variance using SAS^®^ (SAS Institute, Inc., Cary, NC, USA). Results for multiple comparisons using Duncan’s Multiple Range Test were considered significant at *p* < 0.05. The Kruskal-Wallis test was used when data were not normally distributed (quantification of antiviral response, between different treatments). Means were considered significantly different when *p* < 0.05.

## 3. Results

### 3.1. Low Pathogenic H9N2 AIV Replicates in Chicken CT Mononuclear Cells

**Figure 1:**
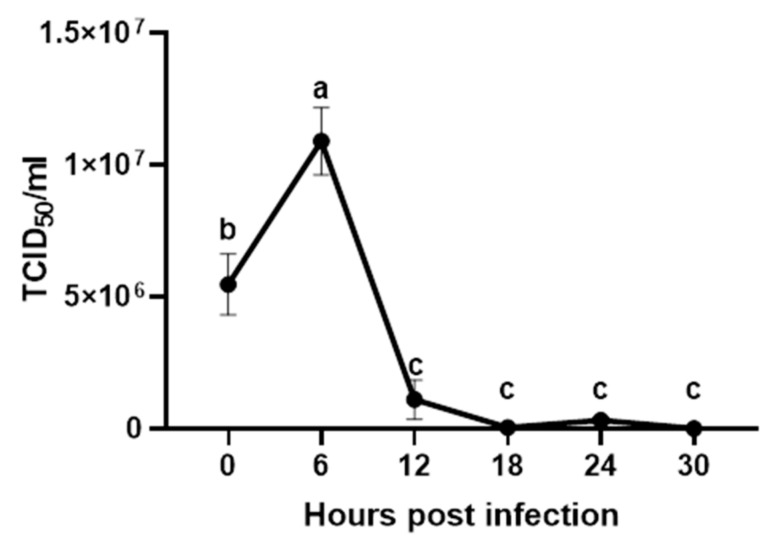
Replication kinetics of low pathogenic H9N2 AIV in chicken cecal tonsils (CT) mononuclear cells. CT mononuclear cells from specific-pathogen-free chickens (*n* = 8 biological replicates) were infected with low pathogenic avian influenza virus H9N2 at an MOI of one. After one hour, CT mononuclear cells were washed and cultured in complete RPMI 1640 medium supplemented with 2 µg/mL of TPCK-trypsin and then incubated for 30 h at 41 °C at 5% CO_2_. PBS was used as negative control. Cell supernatants were collected at zero, six, 12, 18, 24, and 30 h post-infection, and viral titers were determined using MDCK cells. Virus titers are represented as 50% tissue culture infectious dose (TCID_50_/mL). Error bars represent standard errors of the mean, and different letters denote significant differences between time points (*p* < 0.05).

Infectious virus in cell culture supernatants was quantified using the TCID_50_ assay for the analysis of viral infection and replication. No virus replication was observed in the non-infected negative control cells, while H9N2 LPAIV-infected cells had viral replication levels across time points post-infection. A dose-dependent increase of H9N2 LPAIV titer was observed in the supernatants of cells infected with different MOI (0.01, 0.1, and 1), and the MOI of one had the highest infection rate in CT mononuclear cells. We therefore chose to use the MOI of one for all subsequent infections in CT mononuclear cells. The results also demonstrate a moderate but significant increase in the virus titer at 6 hpi (1.09 × 10^7^ TCID_50_/mL) compared to the time of infection at 0 hpi (5.47 × 10^6^ TCID_50_/mL). Virus titer was then significantly decreased at 12, 18, 24, and 30 hpi compared to 6 hpi (Figure 1).

### 3.2. Stimulation of Chicken CT Mononuclear Cells with Probiotic Lactobacilli Alone or in Combination with CpG ODN Limits H9N2 LPAIV Replication 

**Figure 2:**
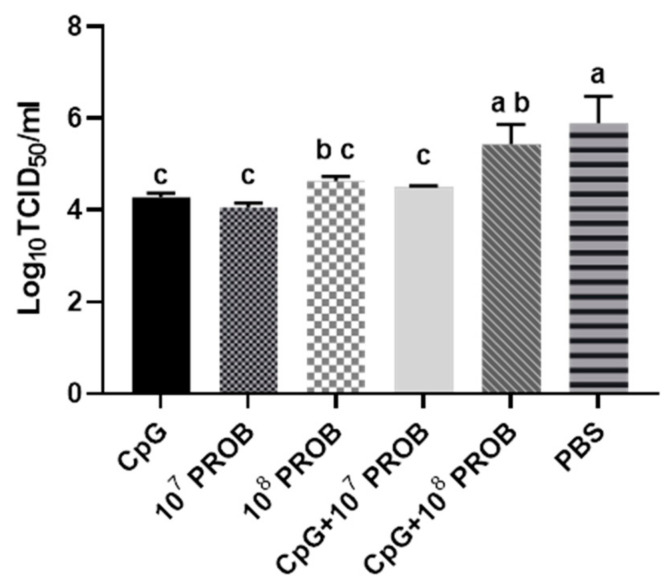
Evaluation of antiviral activity of CT mononuclear cells after stimulation with a probiotic lactobacilli cocktail and/or CpG ODN 2007. CT mononuclear cells from chickens (*n* = 8 biological replicates) were stimulated with a probiotic lactobacilli cocktail (*L. salivairus, L. johnsonii, and L. reuteri*) and/or CpG ODN 2007. Following stimulation for 24 h, cell culture supernatants were removed, and cells were subsequently infected with H9N2 AIV at a MOI of one for one hour. CT mononuclear cells were then washed and suspended in complete RPMI-1640 medium supplemented with 2 µg/mL of TPCK-trypsin. Cells were incubated at 41 °C and 5% CO_2_ for 6 hpi. Cell culture supernatants were collected for virus titration on MDCK cells. Virus was tittered using the 50% tissue culture infectious dose assay and represented as the log_10_ TCID_50_/mL. Standard errors of the means are shown. Bars with different letters were significantly different from each other (*p* < 0.05). Treatment groups were: 5 µg/mL CpG ODN 2007 (CpG), 10^7^ CFUs of lactobacilli (10^7^ PROB), 10^8^ CFUs of lactobacilli (10^8^ PROB), CpG+10^7^ PROB, CpG+10^8^ PROB and phosphate-buffered saline (PBS).

To evaluate of antiviral activity of lactobacilli in CT cells, cells were stimulated ex vivo with different treatments, including CpG, lactobacilli in two concentrations, combinations of CpG with two different concentrations of lactobacilli or PBS. After 24 h of stimulation, cells were washed and infected with H9N2 AIV at a MOI of one for 6 h. As depicted in Figure 2, mononuclear cells treated with CpG, 10^7^ CFU of lactobacilli or CpG with 10^7^ CFU of lactobacilli had the most significant reduction of virus titers compared to the PBS-treated negative control group (*p* < 0.05). No differences were observed in the virus titers between the 10^8^ PROB and CpG+10^8^ PROB groups. The viral titers in the CpG+10^8^ PROB group were not significantly different from the PBS group. 

### 3.3. Stimulation of Chicken CT Mononuclear Cells with Probiotic Lactobacilli Alone or in Combination with CpG ODN Did not Induce NO Production by CT Cells

**Figure 3:**
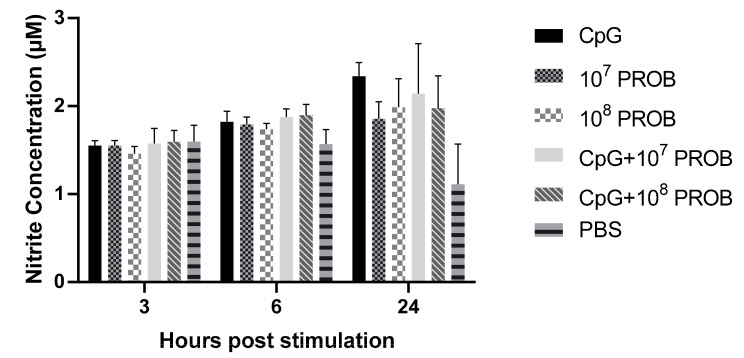
Nitric oxide (NO) production by CT mononuclear cells post-stimulation with lactobacilli and/or CpG. CT mononuclear cells from chickens (*n* = 8 biological replicates) were stimulated with a probiotic lactobacilli cocktail (*L. salivairus, L. johnsonii, and L. reuteri*) and/or CpG ODN 2007 for 3, 6, and 24 hs. PBS or RPMI 1640 was used as vehicle control. At each time point, supernatants were collected and used in a Griess assay for quantification of NO production by CT mononuclear cells. Error bars represent standard errors of the means. Treatment groups were: 5 µg/mL CpG ODN 2007 (CpG), 10^7^ CFUs of lactobacilli (10^7^ PROB), 10^8^ CFUs of lactobacilli (10^8^ PROB), CpG+10^7^ PROB, CpG+10^8^ PROB and phosphate-buffered saline (PBS).

To investigate the mechanism by which probiotic lactobacilli affect CT cells, we stimulated CT cells with different concentrations of lactobacilli alone or in combination with CpG ODN. We then measured NO in supernatants of CT cells. NO is formed by an initial stimulation of expression of inducible NO synthase. NO is a well-known endogenously produced molecule that can have both stimulatory and suppressive effects on the immune system [42,43]. Our result demonstrates that stimulation of CT mononuclear cells with lactobacilli, alone or in combination with CpG ODN, did not affect NO production when compared to PBS-treated cells at all time points (Figure 3).

### 3.4. Stimulation of Chicken CT Mononuclear Cells with Probiotic Lactobacilli Induces Cytokine, Chemokine, and Antiviral Gene Expression

**Figure 4:**
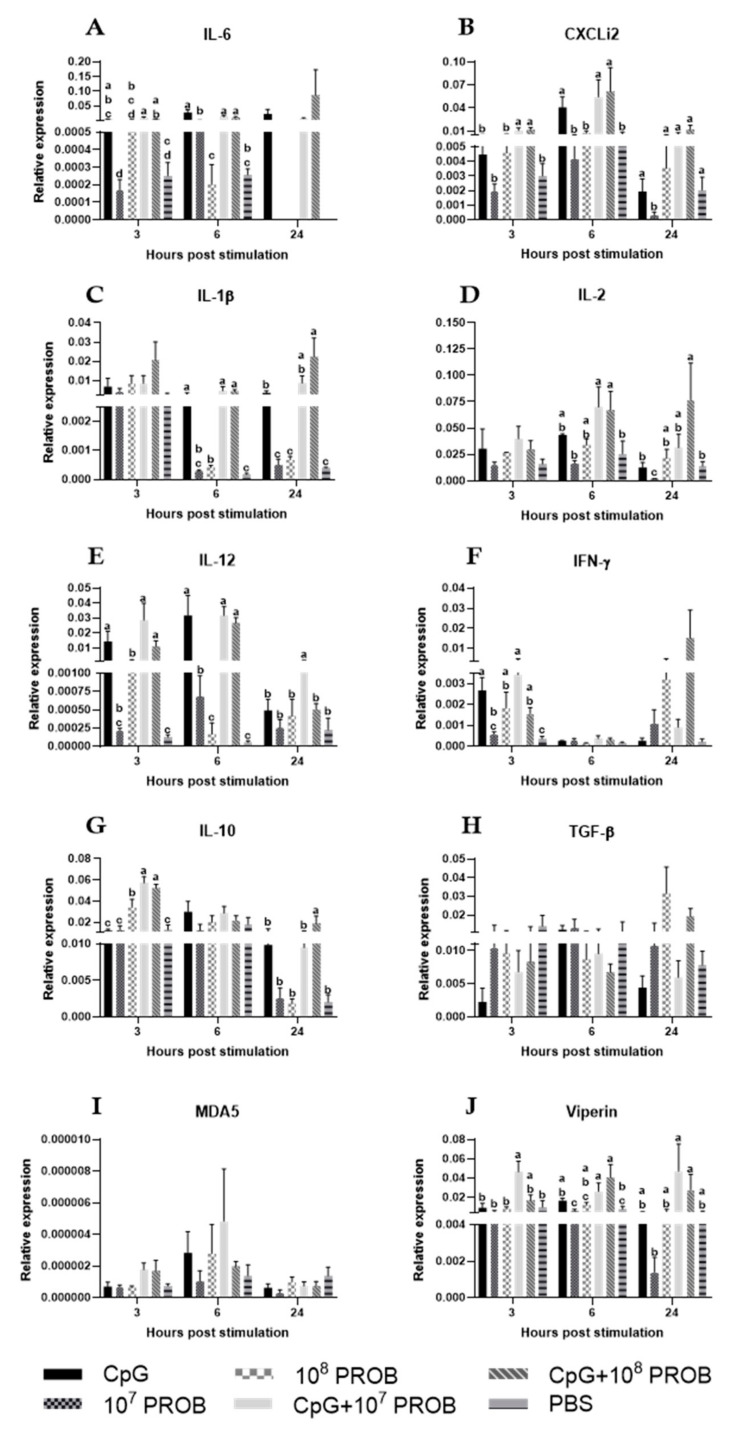
Relative expression of cytokine, chemokine, and antiviral genes in chicken CT mononuclear cells after stimulation with lactobacilli and/or CpG. CT mononuclear cells from chickens (*n* = 8/treatment group) were stimulated with a probiotic lactobacilli cocktail (*Lactobacillus salivairus, L. johnsonii, and L. reuteri*) and/or CpG ODN 2007 for 3, 6, and 24 h. PBS or RPMI 1640 were used as vehicle controls. At each time point, CT mononuclear cells were collected in TRIzol^®^ for RNA extraction. Because of low RNA yield, RNA samples were pooled after RNA extraction. There were four biological replicates/treatment group/time point. After cDNA synthesis and qRT-PCR, the expression levels of target genes, as well as the housekeeping gene (β-actin), were determined. The expression levels are shown as relative to β-actin. Error bars indicate standard error of the mean of four biological replicates. Different letters indicate a significant difference between different treatments within a time point (*p* < 0.05). Treatment groups were: 5 µg/mL CpG ODN 2007 (CpG), 10^7^ CFUs of lactobacilli (10^7^ PROB), 10^8^ CFUs of lactobacilli (10^8^ PROB), CpG+10^7^ PROB, CpG+10^8^ PROB and phosphate-buffered saline (PBS).

At 3 hps, treatment of mononuclear cells with CpG+10^7^ PROB or CpG+10^8^ PROB induced significantly higher expression of interleukin (IL)-6 and CXCLi2 compared to PBS-treated cells (Figure 4A,B). Expression of IL-12p40 and IFN-γ genes were higher in groups treated with CpG, 10^8^ PROB, or CpG in combination with either concentration of lactobacilli compared to the PBS-treated cells (Figure 4E,F). Treatment with CpG in combination with either concentration of lactobacilli induced significantly higher expression of IL-10 compared to all other groups (Figure 4G), while treatment with CpG in combination with 10^7^ PROB induced the highest gene expression of viperin (Figure 4J). We did not observe significant differences in gene expression of IL-1β, IL-2, transforming growth factor-beta (TGF-β) or melanoma differentiation-associated protein 5 (MDA5) among all treatments at 3 hps (Figure 4C,D,H,I, respectively).

At 6 hps, cells treated with CpG or CpG in combination with either concentration of lactobacilli had significant gene expression of IL-6, CXCLi2, IL-1β, and IL-12p40 compared to other treatments (Figure 4A–C,E, respectively). Treatment with CpG in combination with either concentration of lactobacilli induced higher gene expression of IL-2 and viperin compared to PBS treatment (Figure 4D,J). No significant differences were observed in IFN-γ, IL-10, TGF-β, and MDA5 among all treatments at this time point (Figure 4F,G,H,I, respectively).

At 24 hps, treatment of mononuclear cells with 10^7^ PROB downregulated gene expression of CXCLi2 and IL-2; in contrast, treatment with CpG+10^8^ PROB showed increased expression of IL-2 compared to both CpG and PBS treatments (Figure 4B,D, respectively). Treatment with CpG+10^7^ PROB induced significantly higher gene expression of IL-12p40, while treatment with CpG+10^8^ PROB induced significantly higher expression of IL-10 compared to all other treatments (Figure 4E,G). IL-1β gene expression was higher in cells treated with CpG+10^8^ PROB compared to CpG- and PBS-treated cells (Figure 4C). Treatment with CpG in combination with either concentration of lactobacilli induced higher expression of viperin compared to 10^7^ PROB treatment (Figure 4J). No significant differences were observed in gene expression of IL-6, IFN-γ, TGF-β, and MDA5 among all treatments at this time point (Figure 4A,F,H,I, respectively).

## 4. Discussion

There is accumulating evidence pointing to the association between GALT, the gut microbiome and response to viruses. In a recent series of experiments, H9N2 AIV infection in chickens was shown to disrupt the composition of intestinal microbiota, which made the host susceptible to higher viral replication and consequently to higher virus shedding [2,35,44]. Cecal tonsils are a major lymphoid tissue of avian GALT. CTs appear in chicken embryos as early as ten embryonic incubation days (EID), and lymphocytes can be detected by 18 EID [45]. The unique multicellular composition of cecal tonsils that includes a specialized lymphoepithelium, a heterogeneous population of B and T cells and mononuclear phagocytes, makes it a potential target for induction of both innate and adaptive immune responses against H9N2 AIV infections of the chicken gut [45].

In chickens, *Leuconostoc mesenteroides* YML003 showed antiviral activity against H9N2 LPAIV in vitro and in vivo [46]. Similarly, *Lactobacillus plantarum* has an immunomodulating effect against the H9N2 virus [47]. Additionally, a study by our group has demonstrated the impact of depletion of gut microbiota by antibiotics and reconstitution of the microbiome by fecal transplantation on immunity to H9N2 virus [35,36]. Altogether, these studies underscore the importance of the relationship between commensal microbes of the gut, the host immune system and, ultimately, immunity against invading pathogens.

In the present study, we found that CTs provided a replication niche for H9N2 LPAIV, as demonstrated by replication of this virus in CT mononuclear cells in vitro. These findings triggered us to investigate whether pre-treatment with probiotic lactobacilli could elicit innate and antiviral responses in CT that can effectively inhibit AIV replication in these cells. Hence, in this study, we used a cocktail of three avian *Lactobacillus* species, including *Lactobacillus salivairus, L. johnsonii, and L. reuteri*, for their immunomodulatory effects on various lymphoid organs and immune system cells, including CT mononuclear cells [18,34]. CpG has shown a significant impact on expression of immune-related genes in vitro [21]; thus, we also sought to investigate whether a combination of lactobacilli and CpG would have additive or synergistic antiviral and immunostimulatory effects. The results of this study revealed that regardless of the concentration, treatment of CT cells with probiotic lactobacilli cocktail alone or in combination with CpG ODN reduced AIV replication. This result could be attributed to the immunostimulatory capability of probiotic lactobacilli that induced CT cells to mount an effective immune response against AIV.

Therefore, we further investigated the mechanisms by which these probiotics induced antiviral responses in CT cells post-stimulation with probiotic lactobacilli. Given the important role of NO in host defense against invading pathogens, including viruses, bacteria, and protozoa [42,43,48,49], we assessed the effects of the lactobacilli cocktail on NO production by CT mononuclear cells. Indeed, there is still controversy over the antiviral activity of NO against AIV infections. Some studies have demonstrated the potential for NO to inhibit influenza virus replication, while others showed that NO contributes to the pathogenic outcome of influenza virus infections [50,51,52,53]. In this study, neither CpG ODN nor probiotics or their combination were able to elicit NO production in CT mononuclear cells. Nonetheless, these results must be interpreted with caution as the inability of these cells to produce NO could be due to the small number of macrophages in the single-cell suspension of CT or the counteracting effects of the different *Lactobacillus* spp. in the mixture. The findings of a recent study support this assumption; *L. salivarius* and *L. johnsonii* enhanced the production of NO in chicken macrophages, whereas *L. reuteri* significantly reduced it [34].

We also evaluated expression of genes associated with T helper type 1 (Th1) responses (IL-2 and IFN-γ) because of their critical role in host defense against intracellular microbial agents and viruses. Activated Th1 cells are the primary source for IL-2, an inflammatory cytokine, which drives differentiation of naïve T cells into effector T cells [54]. IFN-γ is a potent activator of macrophages with strong antiviral properties [55,56]. It has been shown that IFN-γ inhibits viral replication through different mechanisms such as induction of indoleamine 2,3-dioxygenase and nitric oxide synthase [57,58]. The results of the present study demonstrated that regardless of the concentration and time point, the combination of CpG and lactobacilli enhanced IL-2 and IFN-γ expression. In agreement with our results, Yitbarek et al. showed that administration of the same species of probiotic lactobacilli to chickens with depleted intestinal microbiota restored the IFN-γ transcription level in the spleen [36].

Given the important role of pro-inflammatory cytokines and chemokines in the initiation of the inflammatory response that helps control virus proliferation [18], we measured the expression of IL-12p40, IL-6, CXCLi2, and IL-1β in CT cells following exposure to probiotic lactobacilli alone or in combination with CpG ODN. The results revealed that lactobacilli treatment alone was able to induce higher IL-1β and IL-12 expression, while when combined with CpG ODN, significantly enhanced IL-12, IL-6, CXCLi2, and IL-1β expression, as compared to negative controls. It is still unclear, however, whether this is due to additive effects or a dominant effect mediated by CpG ODN as a significant expression of these cytokines was observed in cells treated with CpG ODN alone. Nonetheless, lactobacilli alone demonstrated the ability to elicit expression of IL-12, IL-6, and IL-1β. It is, therefore, conceivable that oral administration of a cocktail of probiotic lactobacilli could result in modulation of immune responses in the GALT, ultimately leading to a reduction in viral replication and protection of the host against infection [41].

An immunoregulatory cytokine, IL-10, plays a vital role in maintaining immune homeostasis by shutting down the synthesis of pro-inflammatory cytokines toward the end of the inflammatory process [59,60]. It also plays an essential role in B-cell survival and differentiation [61]. The results here showed that probiotic lactobacilli alone or in combination with CpG induced higher expression of IL-10 in CT cells, which is indicative of their immunoregulatory properties [62].

As part of cellular antiviral mechanisms, the expression of MDA5 and viperin was investigated. With the absence of RIG-I in chickens, chicken MDA5, rather than TLRs, plays a crucial role in the recognition of RNA ligands, including the genome of influenza virus [63,64]. At the same time, chicken MDA5 is not involved in bacterial RNA recognition [65]. Here, MDA5 expression was not affected by any of the treatments. This absence of effect could result from the lack of recognition of RNA from intact, inactivated lactobacilli used in this study. Additionally, it has been reported that aberrant activation of MAD5-MAVS signaling causes autoimmune disorders [66]. Therefore, the unchanged expression of MDA5 by lactobacilli treatments here may further support the observation of their immunoregulatory role in maintaining immune homeostasis.

Viperin is an interferon-stimulated gene that reduces virus replication and shedding and prevents the release of influenza virus particles from infected cells [67,68]. Treatment of CT cells with combinations of CpG and lactobacilli resulted in a higher expression of viperin. In contrast, treatment with either CpG or lactobacilli alone did not result in different induction of viperin gene expression. The observed role of lactobacilli in inducing the expression of viperin indicates a potential antiviral property of lactobacilli bacteria.

Overall, probiotic lactobacilli clearly showed antiviral and immunostimulatory capacities as demonstrated in the current study by their ability in eliciting antiviral responses (interferon-stimulated gene (viperin)) and robust cytokine expression in CT cells, including Th1 type cytokines (IL-2, IL-12, IFN-γ), pro-inflammatory cytokines (IL-1β and IL-6), and an immunoregulatory cytokine (IL-10). Importantly, the concurrent induction of pro- and anti-inflammatory cytokines in lactobacilli-treated cells suggests a role for lactobacilli in the maintenance of homeostasis of the immune system.

## 5. Conclusions

In conclusion, the present study demonstrated that lactobacilli bacteria, either alone or in combination with CpG ODN have the potential to induce and regulate specific immune-related genes in chicken immune system cells as demonstrated by a significant induction of IL-2, IFN-γ, IL-1β, IL-6, and IL-12, and IL-10 transcripts. Furthermore, pre-treatment of cecal tonsil cells with lactobacilli resulted in a significant reduction of H9N2 AIV replication. Considering the immunomodulatory capabilities of probiotic lactobacilli together with the ability of cecal tonsils to mount an antiviral response, the findings of this study suggest the use of probiotic lactobacilli to promote gastrointestinal immunity against H9N2 AIV of chickens. Further investigations of different mechanisms by which these bacteria induce antiviral immunity in CT cells are warranted.

## Figures and Tables

**Table 1:** Primer sequences used for real-time quantitative PCR.

Gene	Primer Sequence (5′-3′) (F = Forward; R = Reverse)	Annealing Temperature (°C)	GenBank Accession Number
IFN-γ	F: TGGCGGCGGGAGGAAAAGTG	60	NM_001030558
	R: CACCGTGCTCCAGCTCAGGC		
IL-1β	F: GTGAGGCTCAACATTGCGCTGTA	64	Y15006
	R: TGTCCAGGCGGTAGAAGATGAAG		
IL-10	F: AGCAGATCAAGGAGACGTTC	55	AJ621614
	R: ATCAGCAGGTACTCCTCGAT		
IL-12p40	F: CCAAGACCTGGAGCACACCGAAG	60	AY262752.1
	R: CGATCCCTGGCCTGCACAGAGA		
IL-2	F: GCAGGGCACGTTCAGGTGGG	58	NM_204153.1
	R: GCCACACAGCCTGGCTCCCT		
IL-6	F: CTGAAGAACTGGACAGAGAG	60	NM_204628.1
	R: CACCAGCTTCTGTAAGATGC		
CXCLi2	F: CTGAAGGTGCAGAAGCAGAG	64	AJ009800
	R: CCAGCTCTGCCTTGTAGGTT		
MDA5	F: GCAAAACCAGCACTGAATGGG	60	GU570144.1
	R: CGTAAATGCTGTTCCACTAACGG		
TGF-β	F: CGGCCGACGATGAGTGGCTC	60	M31160.1
	R: CGGGGCCCATCTCACAGGGA		
Viperin	F: GGAGGCGGGAATGGAGAAAA	60	KY856894.1
	R: CAGCTGGCCTACAAATTCGC		
β -Actin	F: CAACACAGTGCTGTCTGGTGGTA	60	X00182
	R: ATCGTACTCCTGCTTGCTGATCC		
